# Risk factors for breast fibrosis and unfavorable cosmetic outcomes after breast conserving therapy in the contemporary treatment era: a systematic review

**DOI:** 10.1016/j.breast.2026.104707

**Published:** 2026-01-20

**Authors:** M.C.A.W. Notenboom, W.D. Heemsbergen, M. Franckena, L.B. Koppert, M.A.M. Mureau, R.A. Nout, M.B.E. Menke-Pluijmers, F.E. Froklage

**Affiliations:** a Department of Radiotherapy, Erasmus MC Cancer Institute, University Medical Center Rotterdam, the Netherlands; b Department of Surgery, Albert Schweitzer Hospital, Dordrecht, the Netherlands; c Department of Surgery, Erasmus MC Cancer Institute, University Medical Center Rotterdam, the Netherlands; d Department of Plastic and Reconstructive Surgery, Erasmus MC Cancer Institute, University Medical Center Rotterdam, the Netherlands

## Abstract

**Background:**

This systematic review aimed to identify risk factors for fibrosis and unfavorable cosmetic outcomes after breast conserving therapy (BCT) for breast cancer in the light of contemporary oncoplastic surgery and 3D-radiotherapy techniques.

**Methods:**

The systematic literature search was carried out in Embase, Ovid Medline, Cochrane Central Register of Controlled Trials, and CINAHL. Studies published after 2005, reporting two or more risk factors for fibrosis or unfavorable cosmetic outcomes as primary outcomes were eligible for inclusion. Only prospective studies with 100 or more patients analyzed were included. The Quality In Prognosis Studies tool and Cochrane risk-of-bias tool were used to assess risk of bias. Patient, tumor, and treatment-related predictors were identified.

**Results:**

Twelve papers investigating 12.118 patients were identified. Risk factors for both the development of fibrosis and unfavorable cosmetic outcomes were increasing age, larger tumor size, re-resection, poor early cosmetic outcomes before the start of radiotherapy, high boost dose, boost volume per 10 cc, homogeneity-index, dose whole breast irradiation, and adjuvant chemotherapy. No specific risk factors in the setting of BCT with complex oncoplastic surgery techniques or ultra-hypo fractionated radiotherapy were identified in this review.

**Conclusion:**

The risk factors identified in this review are largely similar to those found in 2D-radiotherapy studies; dose homogeneity was additionally identified. Administering chemotherapy before radiotherapy should be considered for patients requiring both treatments. However, the lack of sufficient high-quality data regarding BCT with (complex) oncoplastic surgery techniques and ultra-hypo fractionated radiotherapy schedules address the need for large, multidisciplinary prospective studies with long-term follow-up.

## Highlights

• Systematic review to identify risk factors for fibrosis and cosmetic outcomes.
• Dose homogeneity was identified as one of the risk factors.
• Need for large, multidisciplinary prospective studies with long-term follow-up.

## 1 Background

Of all breast cancer patients, 60–70 % are treated with breast conserving therapy (BCT) [1]. BCT includes breast conserving surgery (BCS) often combined with a sentinel node procedure or axillary lymph node dissection, followed by irradiation of the breast, with or without the axilla, and (neo-)adjuvant systemic therapy if indicated [[2], [3], [4], [5]]. BCT has shown to provide survival outcomes comparable to mastectomy, while allowing patients to preserve their breast [4,6].

Over the past few decades, new oncoplastic surgery and radiotherapy techniques for BCT have been developed and implemented, with the primary objective of improving cosmetic outcomes while maintaining oncological safety [7]. The principles of oncoplastic surgery emerged in Europe during the early 1990s; however, widespread integration into clinical practice occurred only in the late 1990s and early 2000s. From the mid-2000s onward, these oncoplastic surgery procedures achieved broader implementation in the United States and subsequently in other countries worldwide [8,9]. Oncoplastic procedures encompass a range of strategies, from simple oncoplastic surgery techniques including closure of glandular tissue to more complex techniques involving volume displacement and replacement [7,10,11]. Currently, these complex techniques are utilized in approximately 27 % of all BCT cases [12].

Before 2005, radiotherapy was mainly based on two-dimensional (2D) X-ray imaging, limiting accuracy of treatment field definition and dose homogeneity compared to modern three-dimensional (3D) techniques. 3D-conformal radiotherapy (3D-CRT) uses 3D-volumetric imaging techniques, such as a CT scan, for treatment planning, and optimizing treatment accuracy. Intensity Modulated Radiotherapy *(*IMRT) and Volumetric-Modulated Arc Therapy (VMAT) are advanced 3D-radiotherapy techniques, that dynamically adjust radiation beam, intensity and delivery angles, allowing for increased homogeneity and conformity of treatment plans. Moreover, (ultra)hypofractionation has made its advance, using less fractions with a higher dose per fraction, with similar rates of local tumor control and late adverse effects compared to conventionally fractionated schedules [13,14].

Unfortunately, fibrosis of the breast occurs as a late adverse event in a substantial subset of patients after BCT, affecting 10–30 % of patients, with 2–5 % experiencing severe fibrosis [15,16]. Fibrosis can cause pain and can negatively influence cosmetic outcomes and quality of life (QoL) [17]. From different trials using 2D-radiotherapy techniques, it has become evident that the development of fibrosis and poor cosmetic outcomes, and consequently unfavorable QoL, is multifactorial and encompasses patient, tumor, and treatment related risk factors. Reported risk factors for fibrosis are for example age, tumor location (lower quadrants), larger tumor size, larger excision volume, hematoma or seroma after surgery, concomitant chemoradiation therapy, total radiation dose, higher maximum whole breast irradiation dose, and tumor bed boost [15,18]. Reported risk factors for poor cosmetic outcomes are, for example, tumor location (lower quadrants), larger excision volumes, and boost with photons [19,20].

It is unclear whether established risk factors for fibrosis also apply to the contemporary techniques used, i.e. simple (with closure of glandular tissue) or complex oncoplastic surgery and modern 3D-radiotherapy techniques. Therefore, this systematic review aimed to identify risk factors, which are related to fibrosis and unfavorable cosmetic outcomes after BCT, with a focus on contemporary used oncoplastic surgery and radiotherapy techniques.

## 2 Methods

### 2.1 Search strategy

This systematic review was registered at PROSPERO (ID: CRD42022298749) and was performed in accordance with the PRISMA guidelines [21]. A systematic literature search was carried out in January 2025 in Embase, Ovid Medline, Cochrane Central Register of Controlled Trials, and CINAHL. The search terms, medical subject headings (MeSH) terms, and synonyms are shown in Supplementary material A.

### 2.2 Eligibility criteria

Only prospective studies and randomized controlled trials reporting on patient, tumor, and treatment-related factors associated with fibrosis of the breast or cosmetic outcomes, as primary outcome in the context of BCT were eligible for inclusion. All studies using 2D-radiotherapy with or without oncoplastic surgery were excluded. Studies with a follow-up period of less than one year were excluded. Furthermore, publications reporting the use of breast implants, partial breast irradiation, re-irradiation, brachytherapy, intra-operative radiotherapy, radiotherapy with protons, and patients with metastasized breast cancer were excluded. Studies investigating fewer than two potential risk factors or without multivariable analyses were excluded, because risk factors may be confounders. Retrospective studies and prospective studies with less than 100 patients were excluded, because of a lower level of evidence. If multiple papers analyzing the same cohort were eligible for inclusion, only the initial study was included.

### 2.3 Selection process

Two reviewers (MN, FF) independently screened all potentially eligible papers for title and abstract taking the eligibility criteria into account. Differences in opinion were resolved by discussion. Subsequently, the full text of each paper was reviewed in accordance with the inclusion and exclusion criteria. In case of any doubt during full text reading, the papers were discussed, and consensus was reached (MN, FF). After the set of studies was established, the data collection process started.

### 2.4 Data collection process

One reviewer (MN) performed the data extraction from the included publications. The data items that were included were year and country of publication, study design, number of patients analyzed, treatment period, toxicity assessment method, and follow-up duration. Furthermore, surgical and radiotherapeutic techniques, whole breast and boost fractionation characteristics, and type of systemic treatment were registered.

### 2.5 Study risk of bias assessment

Risk of bias assessment was performed by one reviewer (MN; Supplementary material B and C) using the Quality In Prognosis Studies (QUIPs) tool for prospective studies and Cochrane risk-of-bias tool for randomized trials [22,23]. The risk of bias was scored as low, medium, or high. Based on the risk of bias assessment, none of the studies were excluded from this review.

### 2.6 Synthesis methods

For synthesis, the papers were grouped into outcome-related groups, namely fibrosis and cosmetic outcomes. Risk factors were categorized into patient, tumor, surgery, radiotherapy, and systemic therapy related risk factors, for which odds and hazard ratios were reported. A meta-analysis could not be performed due to the clinical and methodological heterogeneity of the included studies.

## 3 Results

### 3.1 Study selection

**Fig. 1:**
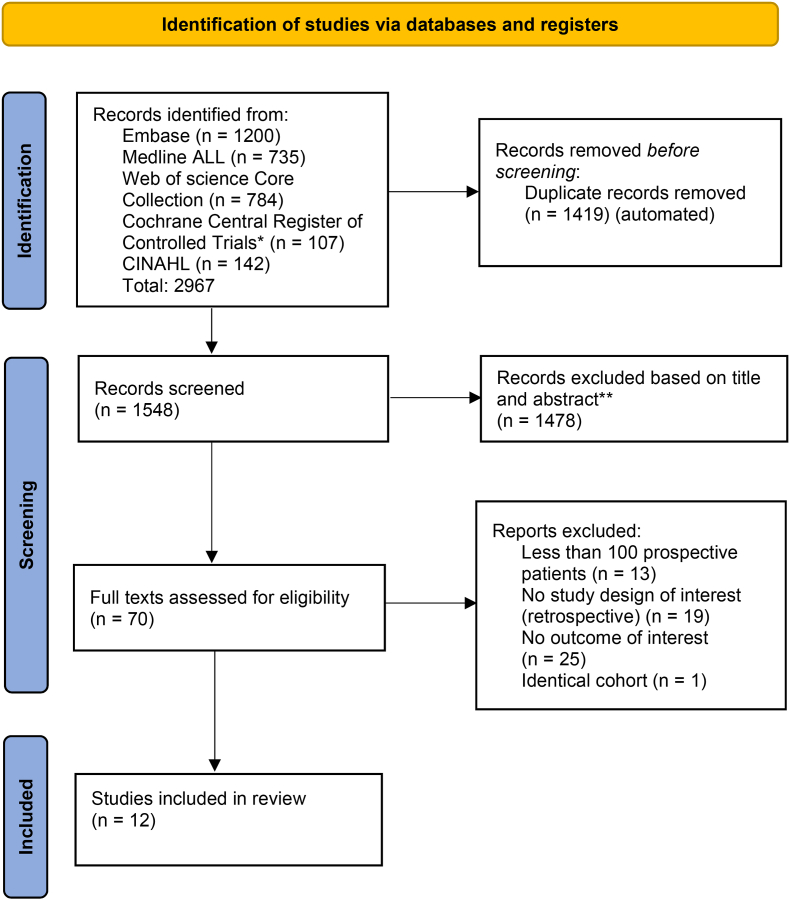
PRISMA flow diagram.

A total of 2967 papers were identified from the initial search strategy in January 2025. After removing duplicates, 1548 papers were screened on title and abstract. Subsequently, 70 papers underwent full text screening, resulting in the inclusion of 12 papers (Fig. 1) [[24], [25], [26], [27], [28], [29], [30], [31], [32], [33], [34], [35]]. Study characteristics are summarized in Table 1. If there was a high risk of bias, it was mostly due to various issues with missing data.

**Table 1:** Overview of the included studies.

Author (year)	Country/cohort	Study design^a^	Treatment period	N	Fibrosis assessment^#^	Cosmetic outcome assessment^#^	Median FU in months (range)
Allali et al. (2022)^1^	France (CANTO, NCT01993498)	Cohort	2012–2017	3480	CTCAE v4.0	NA	36
Bantema et al. (2012)^2^	The Netherlands	Cohort	2005–2010	940	CTCAE v3.0	4-point Harris scale	30 (6–54)
Barnett et al. (2011)^3^	United Kingdom	RCT	2003–2007	1014	3-point scale	3-point scale	24
Brouwers et al. (2018)^4^	32 institutes in Europe (18 institutes from The Netherlands, France, Germany NCT00212121)	RCT	2004–2011	2421	4-point Harris scale	BCCT.core software, 4-point Harris scale, Patient's questionnaire	51
Colciago et al. (2021)^5^	Italy	Cohort	2009–2020	425	RTOG scale	NA	78 (67–89)
De Santis et al. (2016)^6^	Italy	Cohort	2009–2014	537	RTOG scale	NA	32
Dewan et al. (2018)^7^	India	Cohort	2014–2017	233	RTOG scale	4-point Harvard scale	18 (12–48)
Hammer et al. (2017)^8^	The Netherlands	Cohort	2005–2009	546	CTCAE	NA	54 (47–61)
Lilla et al. (2007)^9^	Germany	Cohort	1998–2001	416	RTOG/EORTC scale, LENT-SOMA scale	NR	51 (36–77)
Linares et al. (2016)^10^	Spain	Cohort	2006–2011	143	CTCAE v4.0	4-point scale	36
Offersen et al. (2020)^11^	Denmark, Germany, Norway (NCT00909818)	RCT	2009–2014	1854	4-point Harris scale	4-point Harris scale	21.78
Wang et al. (2020)^12^	United States	Cohort	2010–2016	109	NA	Photographs of the breasts (BRA)	12

# Assessment by a physician unless stated otherwise. N = number of patients in analyses; NA = not analyzed; NR = not reported; FU = follow-up duration; CTCAE = Common Terminology Criteria for Adverse Events; RTOG = Radiation Therapy Oncology Group; LENT-SOMA = Late effects of Normal Tissue-Subjective Objective Management Analytical; BRA = breast retraction assessment.

a All included studies were prospective.

### 3.2 Study characteristics

The three included randomized controlled trials and nine prospective cohort studies were published between 2007 and 2022 (Table 1). In total, 12.118 patients were analyzed with a median follow-up time varying from 12 to 78 months. Different scales and grading systems for fibrosis and cosmetic outcome assessment were used throughout the studies, i.e. CTCAE, RTOG scale, 4-point Harris scale, 4-point Harvard scale, 3-point scale, and LENT SOMA scale. Types of BCS that were mentioned in the included studies were: lumpectomy, wide local excision, quadrantectomy, and tumorectomy, but most studies only reported BCS without further specification. Only one study reported that oncoplastic surgery was allowed, however, without further specification [34]. All included studies treated patients with 3D-CRT or IMRT radiotherapy techniques with or without a boost (Table 2). Nodal irradiation protocols were not always specified, and in one study it was not allowed. Radiotherapy fractionation in the included studies were moderately hypofractionated, i.e. 40-42.4 Gy in 15 or 16 fractions and/or conventional schedules of 25 fractions of 2 Gy, with or without a boost (Table 2). None of the included studies used the ultra-hypofractionated (UHF) protocol (5 fractions of 5.2 Gy) [14]. The administration of systemic therapy was not always specified.

**Table 2:** Overview of type of surgical and radiotherapeutic techniques, radiation dose fractionation schedules and type of systemic treatment.

Author (year)	Surgical technique	Radiotherapeutic technique	Whole breast irradiation (fractions x dose per fraction in Gy)	Boost (fractions x dose per fraction in Gy)	Nodal irradiation	Systemic treatment
Allali (2022)^1^	Breast conservative surgery	3D-CRT and IMRT	25 x 2 or 15 x 2.67	8 x 2	NR	Adjuvant chemotherapy, anti-HER2neu, hormone therapy
Bantema (2012)^2^	Lumpectomy	3D-CRT-SIB	28 x 1.8	SIB 28 x 2.3/2.4	Regional RT was allowed	Chemotherapy (FEC sometimes combined with taxanes), anti- HER2neu: (trastuzumab), Endocrine therapy (tamoxifen, or aromatase inhibitors)
Barnett (2011)^3^	Breast conservative surgery	IMRT	15 x 2.67	3 x 3	Regional RT was allowed	Chemotherapy (yes/no), Endocrine therapy (tamoxifen)
Brouwers (2018)^4^	Wide local excision	3D-CRT	25 x 2	Randomized: 16 Gy or 26 Gy	Regional RT was allowed	Chemotherapy, endocrine therapy
Colciago (2021)^5^	Quadrantectomy	3D-CRT	16 x 2.65	4 x 2.5 and 8 x 2	NR	NR
De Santis (2016)^6^	Quadrantectomy	3D-CRT	16 x 2.65	Sequential 4 x 2.5 and 8 x 2	NR	Chemotherapy (Adriamicin, Taxol, CMF), anti- HER2neu (Trastuzumab), endocrine therapy (yes/no)
Dewan (2018)^7^	Breast conservative surgery	IMRT-SIB	28 x 1.64	28 x 2.14	28 x 1.64	Chemotherapy (yes/no), endocrine therapy (trastuzumab)
Hammer (2017)^8^	Lumpectomy	3D-CRT-SIB	28 x 1.8	SIB 28 x 2.3/2.4	28 x 1.8	Chemotherapy (yes/no), anti-HER2neu (Trastuzumab), endocrine therapy (tamoxifen, or aromatase inhibitors)
Lilla (2007)^9^	Breast conservative surgery	3D-CRT	25 x 2 or 28 x 1.8 or 28 x 2	Sequential 5–20 Gy	NR	Chemotherapy (yes/no), endocrine therapy (yes/no)
Linares (2016)^10^	Tumorectomy/lumpectomy or quadrantectomy	3D-CRT	16 x 2.65	SIB 16 x 0.48 or 16 x 0.75 Sequential: 5–8 x 2	16 x 2.65	Chemotherapy (epirubicin and cyclophosphamide), anti-HER2neu (Trastuzumab, endocrine therapy (tamoxifen, aromatase inhibitors)
Offersen (2020)^11^	Breast conservative surgery without immediate reconstruction. Oncoplastic surgery was allowed.	3D-CRT	25 x 2 or 15 x 2.67	Sequential 5–8 x 2	Regional RT was not allowed	Chemotherapy (taxane-based), anti-HER2neu (Trastuzumab), endocrine therapy (tamoxifen, letrozole)
Wang (2019)^12^	Breast-conserving surgery	3D-CRT	25 x 2 or 15 x 2.66	Sequential 5–8 x 2 or SIB total 48 Gy, 0.54 Gy per fraction	25 x 2 or 15 x 2.66	Chemotherapy therapy (yes/no), endocrine therapy (yes/no) and HER2-directed therapies (yes/no)

NR = not reported; Gy = gray, 3D-CRT = 3-dimensional conformal radiation therapy; IMRT = intensity-modulated radiation therapy; SIB = simultaneously integrated boost; HER2 = human epidermal growth factor receptor 2; FEC: 5-fluorouracil, epirubicine, cyclofosfamide; CMF: ciclofosfamide, methotrexate, fluorouracil.

### 3.3 Results of individual studies

**Fig. 2:**
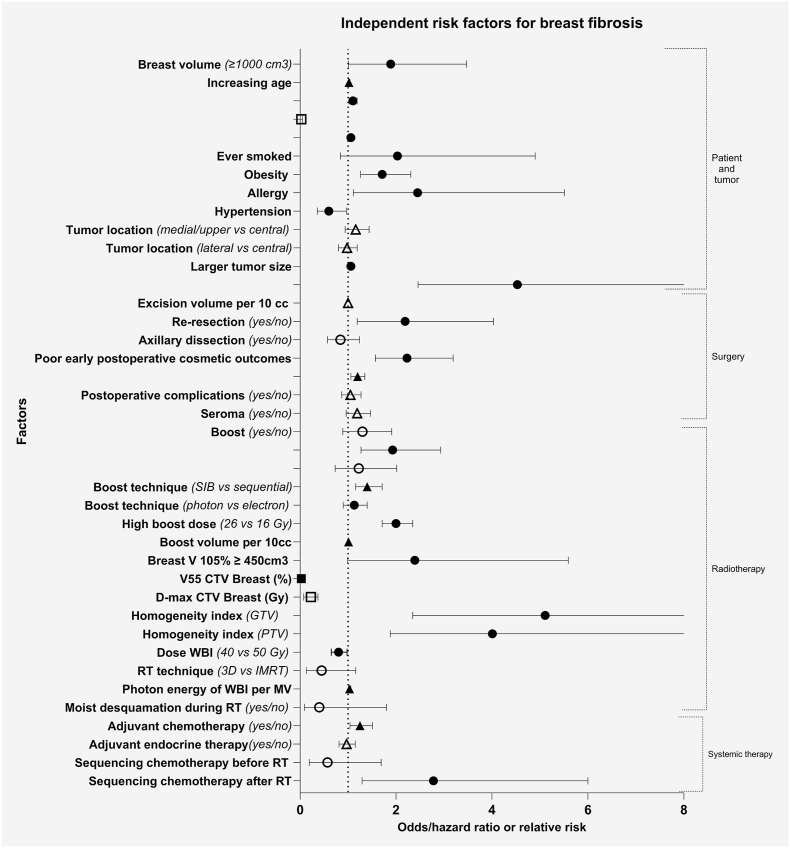
Odds/hazard ratios and relative risks including 95%-confidence intervals of risk factors for fibrosis. Circles represent odds ratios, triangles represent hazard ratios and squares represent relative risks. Filled shapes represent statistically significant findings (p < 0.05), unfilled shapes represent non-significant findings. All ratios are derived from multivariable analyses and include both statistically significant and not significant risk factors. SIB = simultaneously integrated boost; Breast V 105 % = volume of the breast receiving 105 % of the prescribed dose; V55 CTV breast = volume of breast clinical target volume receiving *≥*55 Gy; CTV breast = breast clinical target volume; D-max = maximum dose; GTV = gross tumor volume; PTV = planning target volume; 3D = 3-dimensional conformal radiation therapy; IMRT = intensity-modulated radiation therapy; WBI = whole breast irradiation; Gy = gray; MV = megavoltage; RT = radiotherapy.

**Fig. 3:**
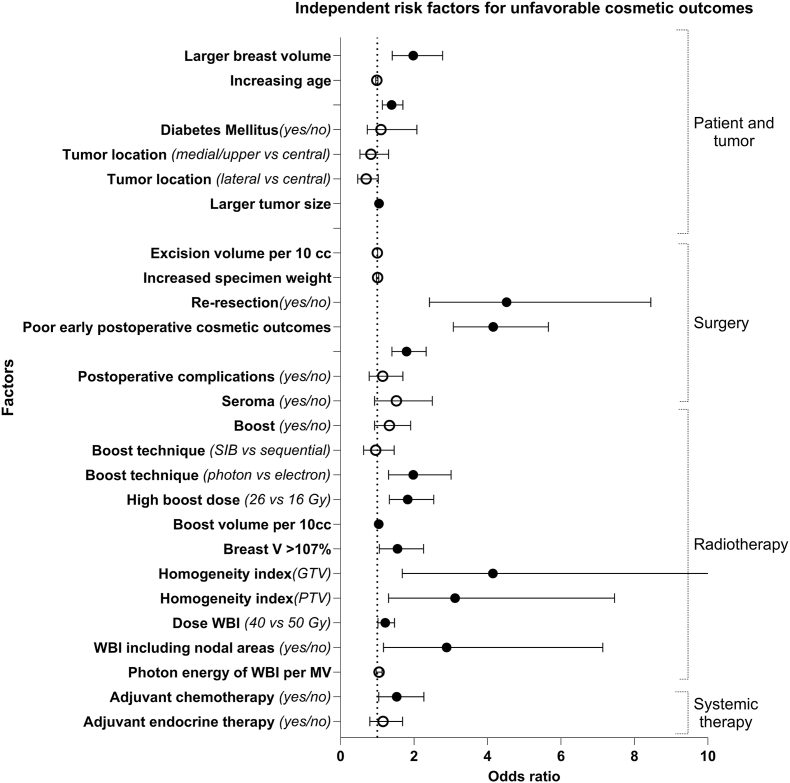
Odds ratios including 95%-confidence intervals of risk factors for unfavorable cosmetic outcomes. Filled circles represent statistically significant findings (p < 0.05), unfilled circles represent non-significant findings. All ratios are derived from multivariable analyses and include both statistically significant and not significant risk factors. SIB = simultaneously integrated boost; Breast V >107 % = volume of the breast receiving >107 % of the prescribed dose; GTV = gross tumor volume; PTV = planning target volume; WBI = whole breast irradiation; Gy = gray; MV = megavoltage.

Seven studies reported on both fibrosis and cosmetic outcomes [[25], [26], [27],^30^,[32], [33], [34]]. Four studies reported only on fibrosis [24,28,29,31] and one study only on cosmetic outcomes [35]. Different potential patient-, tumor-, and treatment-related risk factors for the development of fibrosis or unfavorable cosmetic outcomes were identified in various studies as shown in Fig. 2, Fig. 3, respectively. Table 3 presents an overview of the individual study results of the investigated (potential) risk factors for both the development of fibrosis and unfavorable cosmetic outcomes.

**Table 3:** Overview of investigated risk factors in multivariable analyses for fibrosis and unfavorable cosmetic outcomes.

		Fibrosis		Unfavorable cosmetic outcome	
Factors	Author	Odds/Hazard ratio (95 % CI)	p-value	Odds/Hazard ratio (95% CI)	p-value
Patient and tumor related Breast volume *(≥*1000 cm^3^*)* Larger breast volume Increasing age Ever smoked Obesity *(yes/no)* Allergy *(yes/no)* Hypertension *(yes/no)* Diabetes Mellitus *(yes/no)* Tumor location (*medial/upper vs central)* Tumor location *(lateral vs central)* Larger tumor size	Colciago Barnett Wang Brouwers Dewan Hammer Lilla Lilla Allali Lilla Colciago Barnett De Santis Brouwers Brouwers Bantema Dewan	OR: 1.89 (1.01, 3.47) – – HR: 1.02 (1.01, 1.04) OR: 1.10 (1.02, 1.19) RR: 0.026 (−0.001, 0.053) OR: 1.06 (1.01, 1.11) OR: 2.03 (0.84, 4.90) OR: 1.71 (1.26, 2.31) OR: 2.45 (1.11, 5.51) OR: 0.60 (0.36–0.97) – OR: 1.2 (NR) HR: 1.16 (0.94, 1.44) HR: 0.98 (0.80, 1.19) OR: 1.06 (1.03, 1.09) OR: 4.53 (2.46, 8.35)	0.042 – – 0.005 0.017 0.054 0.03 NS <0.007 NR 0.041 – 0.52 0.17 0.081 <0.001 <0.001	– OR: 1.98 (1.41, 2.78) NR OR: 0.99 (0.96, 1.02) OR: 1.39 (1.14, 1.70) – – – – – – OR: 2.08 (0.73, 1.10) – OR: 0.83 (0.53, 1.31) OR: 0.70 (0.47, 1.03) OR: 1.05 (1.02, 1.08) OR: 26.9 (5.96, 121.2)	– 0.0005 0.03 0.557 0.001 – – – – – – 0.10 – 0.429 0.073 0.001 <0.001
Surgery related Type of surgery *(lumpectomy vs quadrantectomy)* Excision volume per 10 cc Increased specimen weight Re-resection *(yes/no)* Axillary dissection *(yes/no)* Poor early postoperative cosmetic outcomes Postoperative complications *(yes/no)* Seroma *(yes/no)*	Linares Brouwers Barnett Bantema Allali Barnett Brouwers Brouwers Brouwers	– HR: 1.00 (1.00, 1.00) – OR 2.19 (1.19, 4.03) OR: 0.84 (0.57, 1.24) OR: 2.23 (1.57, 3.19) HR: 1.20 (1.06, 1.35) HR: 1.05 (0.87, 1.27) HR: 1.19 (0.96, 1.47)	– 0.28 – 0.012 0.373 <0.0005 0.003 0.62 0.11	NR OR: 1.00 (1.00, 1.01) OR: 1.01 (0.98, 1.04) OR: 4.52 (2.42, 8.45) – OR: 4.16 (3.07, 5.66) OR: 1.80 (1.40, 2.33) OR: 1.15 (0.78, 1.70) OR: 1.52 (0.93, 2.50)	0.05 0.448 0.73 <0.001 – <0.0005 <0.0001 0.478 0.097
Radiotherapy related Boost *(yes/no)* Boost technique *(SIB vs sequential)* Boost technique *(photon vs electron)* High boost dose *(26 vs*16 Gy*)* High boost dose *(16 vs*10 Gy*)* Boost volume per 10 cc Breast V >107 % Breast V 105 % ≥ 450 cm^3^ V55 CTV Breast (%) D-max CTV Breast (Gy) Homogeneity index *(GTV)* Homogeneity index *(PTV)* RT technique *(3D vs IMRT)* Dose WBI *(50 vs 39.*9 Gy*)* Dose WBI *(42.*5 Gy *vs other)* Dose WBI *(40 vs*50 Gy*)* WBI including nodal areas *(yes/no)* Photon energy of WBI per MV Moist desquamation during RT *(yes/no)*	Allali Barnett Colciago De Santis Brouwers Linares Brouwers Brouwers Wang Brouwers Barnett Colciago Hammer Hammer Dewan Dewan Allali Wang Linares Offersen Bantema Linares Wang Brouwers Lilla	OR: 1.30 (0.89, 1.91) OR: 1.93 (1.27, 2.93) OR: 1.22 (0.73, 2.02) OR: 1.5 (NR) HR: 1.40 (1.16, 1.71) – HR: 1.13 (0.90, 1.40) HR: 2.00 (1.71, 2.35) – HR: 1.01, (1.01, 1.02) – OR: 2.39 (0.99, 5.59) RR: 0.027 (0.007, 0.046) RR: 0.224 (0.075, 0.373) OR: 5.11 (2.35, 11.45) OR: 4.01 (1.88, 8.57) OR: 0.45 (0.13, 1.16) – – OR: 0.80 (0.65, 0.98) – – – HR: 1.03 (1.01, 1.06) OR: 0.40 (0.09, 1.80)	0.172 0.002 0.434 0.24 0.0006 – 0.30 <0.0001 – <0.0001 – 0.047 0.008 0.076 <0.001 <0.001 0.102 – – 0.029 – – – 0.007 NS	– OR: 1.33 (0.93, 1.91) – – OR: 0.96 (0.63, 1.46) NR OR: 1.98 (1.31, 3.01) OR: 1.83 (1.33, 2.54) NR OR: 1.04 (1.02, 1.05) OR: 1.55 (1.06, 2.26) – – – OR: 4.15 (1.68, 10.25) OR: 3.12 (1.31, 7.46) – NR NR OR: 1.22 (1.02, 1.47) OR: 2.89 (1.17, 7.14) NR NR OR: 1.05 (0.97, 1.13) –	– 0.12 – – 0.837 0.004 <0.0001 <0.0001 0.046 <0.0001 0.023 – – – 0.002 0.010 – 0.02 0.001 0.032 0.02 0.5 0.02 0.232 –
Systemic therapy related Adjuvant chemotherapy *(yes/no)* Adjuvant endocrine therapy *(yes/no)* Sequencing chemotherapy before RT Sequencing chemotherapy after RT	Brouwers De Santis Brouwers Bantema Bantema	HR: 1.25 (1.04, 1.51) OR: 1.4 (NR) HR: 0.97 (0.81, 1.15) OR: 0.57 (0.19, 1.69) OR: 2.78 (1.29, 6.00)	0.017 0.26 0.72 0.31 0.009	OR: 1.53 (1.04, 2.27) – OR: 1.16 (0.80, 1.69) – –	0.032 – 0.429 – –

Most studies fitted the endpoint of grade ≥2 fibrosis. All studies fitted the endpoint of fair/poor cosmetic outcomes.

CI = confidence interval; OR = odds ratio; HR = hazard ratio; RR = relative risk; NR = not reported; NS = not significant; SIB = simultaneously integrated boost; Breast V >107 % = volume of the breast receiving >107 % of the prescribed dose; Breast V 105 % = volume of the breast receiving 105 % of the prescribed dose; V55 CTV breast = volume of breast clinical target volume receiving *≥*55 Gy; CTV breast = breast clinical target volume; D-max = maximum dose; GTV = gross tumor volume; PTV = planning target volume; 3D = 3-dimensional conformal radiation therapy; IMRT = intensity-modulated radiation therapy; WBI = whole breast irradiation; Gy = gray; MV = megavoltage; RT = radiotherapy.

### 3.4 Patient and tumor related risk factors

#### 3.4.1 Breast fibrosis

Nine of the included studies reported on different potential patient- and tumor-related risk factors for fibrosis. Larger breast volume (≥1000 cm^3^), increasing age, obesity (yes/no), allergy (yes/no), hypertension (yes/no), and larger tumor size were significantly associated with fibrosis [24,25,27,28,30,32,34]. While most identified risk factors are well-established predictors, the observed associations with allergy and hypertension provide potentially novel insights. Although one study found age to be borderline significant in univariate analysis (p = 0.05), increasing age was ultimately included in their prediction model for fibrosis [31]. Ever smoked, diabetes mellitus (yes/no), and tumor location (medial/upper vs central and lateral vs central) were not found to be significant risk factors for fibrosis in the included studies [27,29,32]. However, one study found that active smokers have a significantly increased risk of fibrosis [34].

#### 3.4.2 Cosmetic outcomes

Five studies investigated potential patient and tumor-related risk factors for unfavorable (fair/poor) cosmetic outcomes. Larger breast volume was significantly associated with unfavorable cosmetic outcomes according to two studies [26,35]. Two other studies identified larger tumor volume as a significant factor for unfavorable cosmetic outcomes [25,30]. One study found increasing age to be associated with unfavorable cosmetic outcomes, whereas in another study there was no significant association [27,30]. Diabetes mellitus (yes/no) and tumor location (medial/upper vs central and lateral vs central) could not be identified as independent risk factors for unfavorable cosmetic outcomes [26,27].

### 3.5 Surgery related risk factors

#### 3.5.1 Breast fibrosis

Four studies reported potential surgery-related risk factors for fibrosis. Re-resection (yes/no) and poor early postoperative cosmetic outcomes (pre-radiotherapy) were significant risk factors for fibrosis [[25], [26], [27]]. Excision volume per 10 cc, axillary dissection (yes/no), post-operative complications (yes/no), and seroma (yes/no) did not appear to be significant risk factors for fibrosis according to the studies that investigated these factors [24,27].

#### 3.5.2 Cosmetic outcomes

Four of the included studies reported on potential surgery-related risk factors for unfavorable cosmetic outcomes and found type of surgery (quadrantectomy instead of lumpectomy), re-resection (yes/no), and poor early postoperative cosmetic outcomes (pre-radiotherapy) to be significant factors [[25], [26], [27],^33^]. Two of these studies also investigated factors that were not significantly associated with unfavorable cosmetic outcomes, i.e. excision volume per 10 cc, increased specimen weight, postoperative complications (yes/no), seroma (yes/no) [26,27]. However, larger excision volume and seroma are generally regarded as factors associated with unfavorable cosmetic outcomes.

### 3.6 Radiotherapy related risk factors

#### 3.6.1 Breast fibrosis

Nine studies reported potential radiotherapy-related factors for the development of fibrosis. Boost technique (simultaneous integrated boost instead of sequential boost), high boost dose, boost volume per 10 cc, a breast volume receiving 105 % or more of the prescribed dose higher than 450 cm^3^ (vs < 450 cm^3^), volume of the clinical target volume (CTV) of the breast receiving more than 55 Gy, homogeneity index of gross tumor volume (GTV) ≥ 0.0506 (vs < 0.0560), homogeneity index of planning target volume (PTV) ≥ 0.296 (vs < 0.296), total dose of whole breast irradiation (WBI) (more fibrosis after 50 Gy than after 40 Gy), and photon energy of WBI per MV were significantly associated with fibrosis [27,28,30,31,34]. Furthermore, only one study found a boost (yes/no) to be a significant factor [26]. In four studies, the use of a boost (yes/no) was not significantly associated with fibrosis [24,28,29,34]. Interestingly, one of these studies was the only one where oncoplastic surgery was allowed [34]. The following evaluated potential factors were also not significantly associated with fibrosis: photon boost (compared to electron boost), increasing maximum radiation dose in the breast (D-max CTV breast), radiotherapy technique (3D vs IMRT), and moist desquamation during radiotherapy (yes/no) [24,27,31,32].

#### 3.6.2 Cosmetic outcomes

Seven studies reported on potential radiotherapy-related factors for the development of unfavorable cosmetic outcomes. Photon boost (instead of an electron boost), high boost dose (26 vs 16 Gy and 16 vs 10 Gy), boost volume per 10 cc (OR 1.04 per 10 cc; median 132 cc, range 0–1308), volume of the breast receiving >107 % of the prescribed dose, homogeneity index of gross tumor volume (GTV) ≥0.0506 (vs > 0.0560), and homogeneity index of planning target volume (PTV) ≥0.296 (vs > 0.296) were significantly associated with unfavorable cosmetic outcomes. One study compared 50 Gy vs 39.9 Gy and one study 40 Gy vs 50 Gy and both studies found better cosmetic outcomes after hypo-fractionated radiotherapy schedules [34,35]. One study found better cosmetic outcomes if patients received 42.5 Gy compared to other schedules (i.e. 40 Gy, 48.5 Gy, 53 Gy, or 55.6 Gy) [33].

One study found worse cosmetic outcomes after a sequential boost (vs simultaneous integrated boost) and another study found no association between the simultaneous integrated boost or sequential boost [27,33]. WBI including nodal areas (yes/no) was an independent risk factor for unfavorable cosmetic outcomes in two studies; however, this was not found in another study [25,33,35]. Other factors that were not significantly associated with unfavorable cosmetic outcomes were boost (yes/no), and photon energy of WBI per MV [26,27].

### 3.7 Systemic therapy related risk factors

#### 3.7.1 Breast fibrosis

Five studies reported on systemic therapy-related risk factors for fibrosis [25,27,29,33,34]. Sequencing chemotherapy after radiotherapy (compared to before radiotherapy) was significantly associated with the development of fibrosis in one study [25]. Adjuvant chemotherapy (yes/no) was significantly associated with the development of fibrosis in one study; however, this was not found in two other studies [27,29,34]. Adjuvant endocrine therapy (yes/no) and sequencing chemotherapy before radiotherapy were not significant risk factors for fibrosis [25,27,34]. Furthermore, one study found that sequencing chemotherapy before or after surgery was not associated with increased risk of fibrosis [33].

#### 3.7.2 Cosmetic outcomes

Adjuvant chemotherapy (yes/no) was significantly associated with unfavorable cosmetic outcomes in one study, this association was not found for endocrine therapy (yes/no) [27].

## 4 Discussion

Most evidence of risk factors for fibrosis and unfavorable cosmetic outcomes after BCT comes from studies before the widespread introduction of 3D-conformal radiotherapy and oncoplastic surgery. To our knowledge, we are the first to have performed an up-to-date systematic review of risk factors of fibrosis and unfavorable cosmetic outcomes after BCT, focusing on contemporary (oncoplastic) surgery and 3D-radiotherapy techniques (3D-CRT, IMRT/VMAT). Unfortunately, studies reporting risk factors for fibrosis and unfavorable cosmetic outcomes after BCT with complex oncoplastic surgery techniques could not be found and were therefore not included in this review.

The identified risk factors for the development of fibrosis and unfavorable cosmetic outcomes overlapped, which is plausible, given the interrelationship between fibrosis and cosmetic outcomes, although not necessarily in a one-to-one association [36]. Regarding patient and tumor related factors, increasing age (per year) and larger tumor size (for every mm or cm increase) were statistically significant risk factors for both fibrosis and unfavorable cosmetic outcomes. The association between increasing age and unfavorable cosmetic outcomes may be explained by the higher prevalence of fatty breast tissue in older (postmenopausal) women [37]. Significant surgery-related risk factors for both fibrosis and unfavorable cosmetic outcomes were performing a re-resection and poor early postoperative cosmetic outcomes before the start of radiotherapy. This highlights the potential value of oncoplastic surgery, as it may reduce re-operation rates and improve postoperative cosmetic outcomes [12]. High boost dose (26 vs 16 Gy and 16 vs 10 Gy), boost volume per 10 cc (OR 1.04 per 10 cc; median 132 cc, range 0–1308), homogeneity index (GTV ≥0.0506 vs < 0.0560, and PTV ≥0.296 vs < 0.296), and dose to the whole breast (WBI, 50 Gy vs 40 Gy) were significant radiotherapy related risk factors for both fibrosis and unfavorable cosmetic outcomes. Also, adjuvant chemotherapy was a significant risk factor for both the development of fibrosis as well as unfavorable cosmetic outcomes. Finally, photon boost (compared to electron boost) was a significant risk factor for unfavorable cosmetic outcomes, and photon energy of WBI per MV was a significant risk factor for development of fibrosis.

As mentioned, we could not find studies that investigated potential risk factors regarding breast fibrosis after complex oncoplastic BCS. Oncoplastic surgery techniques are applied more and more in clinical practice to improve cosmetic outcomes and patient satisfaction without compromising oncological safety [12]. Due to these new techniques, an increasing number of patients are eligible for breast-conserving surgery, leading to a decrease in mastectomies [1,38]. While fat necrosis was not identified as a risk factor for poor cosmetic outcomes in the studies included in this review, it has previously been reported as a potential late effect of breast-conserving oncoplastic surgery [39,40]. Especially patients with scattered fibroglandular or fatty breast tissue are more at risk than patients with dense glandular breast tissue [40].

Interestingly, the included studies did not find seroma, postoperative complications, excision volume per 10 cc, and tumor location to be significant risk factors for development of fibrosis or unfavorable cosmetic outcomes. This is in contrast with older large studies which have found postoperative complications, such as seroma and hematoma, excision volume and tumor location to be risk factors for development of fibrosis or poor cosmetic outcomes [15,20,41,42]. It may be hypothesized that the increased use of simple oncoplastic techniques in combination with contemporary radiotherapy reduces the contribution of these risk factors. However, since these risk factors come from large, randomized trials such as the EORTC ‘boost versus no boost’ trial, we feel these risk factors should still be considered clinically relevant. Nevertheless, it is most likely that oncoplastic surgery was not applied in this trial, highlighting the need to place greater emphasis on more recent studies.

In our review, multiple radiotherapy related factors were associated with the development of fibrosis and unfavorable cosmetic outcomes, and in general there was a large overlap with known risk factors from the 2D-era [15,18,20]. Of all these factors, the boost is probably the most discussed risk factor in the current literature. For example, the large, randomized phase 3 EORTC boost no boost trial from the nineties showed that a radiation boost is an important risk factor for fibrosis [16]. On the other hand, we identified some studies that did not find a boost as a significant risk factor of fibrosis in multivariable analysis [24,28,29,34]. An explanation of Offersen et al. is that a radiotherapy boost was used in a few young patients at low dose (10 Gy). These young patients also received adjuvant chemotherapy. Therefore, postoperative induration had already healed in most patients at randomization, explaining the low odds for induration at baseline in the boost group [34]. Bantema et al. reported that among patients receiving chemotherapy, those who underwent radiotherapy prior to chemotherapy had an approximately fivefold higher risk of developing grade 2 fibrosis compared to those who received chemotherapy first [25]. According to Bantema et al., this may partially be explained by the longer interval between surgery and radiotherapy [25]. In addition, chemotherapy during radiotherapy increased the risk of fibrosis [15]. Based on these findings, administering chemotherapy before radiotherapy should be considered for patients requiring both treatments, which is already common practice.

Larger boost volume is also a known risk factor that can negatively influence cosmetic outcomes, and it seems to be more commonly observed in patients treated with oncoplastic surgery because of larger wound beds [43]. With every 10 cm^3^ increase in boost volume, the risk of breast edema and breast fibrosis increased by 21 % and 12 %, respectively, according to Keleman et al. [44]. This stresses the importance of assessing the interplay between the current use of oncoplastic surgery techniques and radiotherapy.

Known risk factors for fibrosis and unfavorable cosmetic outcomes from studies of the 2D-radiotherapy era largely align with the findings of our review [15,18,20]. However, we additionally identified the homogeneity index as a significant risk factor for both fibrosis and fair/poor cosmetic outcomes [30]. This corroborates the results of other studies, in which IMRT was associated with improved dose homogeneity, compared to conventional 2D-radiation [45,46]. A randomized trial comparing 2D-radiotherapy with 3D-IMRT found significantly lower rates of fibrosis in the IMRT group, while patients in the 2D group were more likely to have changes in breast appearance [45]. Importantly, dose homogeneity is often closely related to breast volume, another established risk factor for fibrosis and poor cosmetic outcomes, because achieving a homogenous radiation plan becomes more challenging in larger breasts [46].

The recent Fast Forward randomized controlled trial used modern 3D-techniques and investigated oncological efficacy and safety of WBI with an ultra-hypofractionated schedule (5 fractions of 5.2 Gy versus 15 fractions of 2.67 Gy). After five years of follow up, the ultra-hypofractionated schedule (i.e. 5 fractions) showed no significant differences compared to the moderately fractionated (i.e. 15 fractions) with regard to normal tissue effects [14]. In this trial, BCS with complex oncoplastic surgery techniques were underrepresented, so no definite conclusions can be drawn for this specific patient population.

Although studies considering biological factors are not included in this review, it is important to note that biological factors can play a role in the development of fibrosis as well. For example, some studies have shown that lower Radiation-Induced Lymphocyte Apoptosis (RILA) values are associated with a higher risk of developing fibrosis of the breast after radiotherapy [[47], [48], [49]]. Over the last decades, different tests and biomarkers have been developed to identify patients who will develop severe late toxicity after radiotherapy. However, these tests do not have a place in daily practice yet.

### 4.1 Strengths and limitations

Strengths of this review are inclusion of only prospective studies that analyzed two or more potential risk factors for fibrosis or unfavorable cosmetic outcomes after BCT, using multivariate analysis and included at least 100 patients, with at least a follow-up period of one year. In total, 12 prospective studies with 12.118 patients were included. Interestingly, the included studies are limited by the lack of data on contemporary oncoplastic surgery techniques. In particular, no study was identified that reported results after complex oncoplastic techniques. Most of the included studies did not specify the type of (oncoplastic) BCS, and some of the included studies applied conventional radiotherapy treatment schedules (i.e. 50 Gy in 25 fractions). Furthermore, the methodology and subsequent results among the included studies were very heterogeneous in terms of toxicity scoring systems (i.e. RTOG, CTCAE, LENT-SOMA) and assessments (i.e. physician scored, photographs, patient questionnaire). Therefore, comparison of the risk factors remains challenging, because most studies have investigated different factors, also using varying methods. Because of this heterogeneity, no meta-analysis could be performed for this systematic review.

Despite these limitations, this review provides a useful contribution to the current literature, because it reveals that the current literature is lacking studies on long-term fibrosis and cosmetic outcomes after complex oncoplastic surgery techniques as well as ultra-hypofractionation in the context of BCT. More research is already carried out in the context of oncoplastic surgery techniques, but the need for more multicenter prospective and comparative studies, investigation of the influence of oncoplastic surgery techniques in combination with contemporary radiotherapy schedules, on fibrosis and long-term cosmetic outcomes of breast cancer patients is warranted [[50], [51], [52]]. Nevertheless, the findings of this systematic review provide an important summary of candidate risk factors to consider in the contemporary treatment era, when developing prediction models for fibrosis and cosmetic outcomes after BCT for the large population of breast cancer survivors.

## 5 Conclusions

To our knowledge, this is the first systematic review identifying risk factors associated with breast fibrosis and poor cosmetic outcomes after BCT focusing on contemporary oncoplastic surgery and 3D-radiotherapy. The identified risk factors for the development of fibrosis and unfavorable cosmetic outcomes were increasing age, larger tumor size, performing a re-resection, poor early postoperative cosmetic outcomes before the start of radiotherapy, high boost dose, boost volume per 10 cc, homogeneity index, dose to the whole breast and adjuvant chemotherapy. The rapid developments in both the surgery and radiotherapy domains call for an up-to-date and ongoing evaluation of late effects. Lack of sufficient data on BCT with ultra-hypofractionation and (complex) oncoplastic surgery techniques calls for large prospective studies, where a multidisciplinary approach is of great importance. In an attempt to address this knowledge gap, research into risk factors for fibrosis and unfavorable cosmetic outcomes of the breast after BCT, especially in the light of the interplay between current oncoplastic surgery and radiotherapy techniques, is currently ongoing in a large multicenter observational cohort study (STARLINGS study, ClinicalTrials.gov ID: NCT05263362).

## CRediT authorship contribution statement

**M.C.A.W. Notenboom:** Writing – original draft, Visualization, Methodology, Investigation, Data curation, Conceptualization. **W.D. Heemsbergen:** Writing – review & editing, Methodology, Conceptualization. **M. Franckena:** Writing – review & editing, Conceptualization. **L.B. Koppert:** Writing – review & editing, Conceptualization. **M.A.M. Mureau:** Writing – review & editing, Conceptualization. **R.A. Nout:** Writing – review & editing, Supervision, Methodology, Conceptualization. **M.B.E. Menke-Pluijmers:** Writing – review & editing, Supervision, Methodology, Conceptualization. **F.E. Froklage:** Writing – review & editing, Supervision, Methodology, Investigation, Conceptualization.

## Ethical approval

All research reported has been conducted in accordance with ethical standards.

## Funding

M.C.A.W.N. received a salary from the BeterKeten foundation (Dutch: Stichting BeterKeten) and a research fund from the 10.13039/100031624Albert Schweitzer Hospital for salary as well. The funders were not involved in the study design, data collection, data analysis and interpretation, manuscript preparation or publication decisions.

## Declaration of competing interest

The authors declare the following financial interests/personal relationships which may be considered as potential competing interests: M.C.A.W. Notenboom reports financial support was provided by BeterKeten foundation (salary). R.A. Nout reports a relationship with Dutch Cancer Society and Dutch Research Council that includes: funding grants. The Department of Radiotherapy, Erasmus MC Cancer Institute has a research collaboration with Elekta AB (Stockholm, Sweden), Accuray Inc., (Sunnyvale, CA, USA) and Varian (Palo Alto, CA, USA). The current research was not funded by any of these companies. If there are other authors, they declare that they have no known competing financial interests or personal relationships that could have appeared to influence the work reported in this paper.

## Data Availability

All data generated during this study are included in this publication.
